# Argonaute2 and Argonaute4 Involved in the Pathogenesis of Kawasaki Disease via mRNA Expression Profiles

**DOI:** 10.3390/children12010073

**Published:** 2025-01-08

**Authors:** Zon-Min Lee, Hui-Chuan Chang, Shih-Feng Liu, Ying-Hsien Huang, Ho-Chang Kuo

**Affiliations:** 1 Department of Pharmacy and Kawasaki Disease Center, Kaohsiung Chang Gung Memorial Hospital, Kaohsiung 83301, Taiwan; zonmin@cgmh.org.tw; 2 Department of Pharmacy, Tajen University, Pingtung 90741, Taiwan; 3 Institute for Translational Research in Biomedicine, Kaohsiung Chang Gung Memorial Hospital, Kaohsiung 83301, Taiwan; elaine11142@cgmh.org.tw (H.-C.C.); m85d@cgmh.org.tw (S.-F.L.); 4 Department of Biotechnology, College of Life Science, Kaohsiung Medical University, Kaohsiung 80708, Taiwan; 5 Department of Respiratory Therapy, Kaohsiung Chang Gung Memorial Hospital, Kaohsiung 83301, Taiwan; 6 Department of Pediatrics and Kawasaki Disease Center, Kaohsiung Chang Gung Memorial Hospital, Kaohsiung 83301, Taiwan; yhhuang@cgmh.org.tw; 7 College of Medicine, Chang Gung University, Taoyuan 33302, Taiwan

**Keywords:** Kawasaki disease, endothelial dysfunction, argonaute

## Abstract

Background: Argonautes (AGOs) are a type of protein that degrade specific messenger RNAs, consequently reducing the expression of a specific gene. These proteins consist of small, single-stranded RNA or DNA and may provide a route for detecting and silencing complementary mobile genetic elements. In this research, we investigated which AGO(s) were involved in Kawasaki disease (KD). Methods and Materials: We obtained mRNA-level gene expression profiles from leukocyte samples that had previously been gathered in another study and uploaded to the NCBI GEO database. The Human Transcriptome Array (HTA 2.0) analysis included 50 children with KD prior to IVIG (KD1), 18 children with KD three weeks post-IVIG (KD3), 18 non-febrile controls (HC), and 18 febrile controls (FC), which were arranged in the quoted publications for all materials and methods in order to collect data. We used the default value of the commercialized microarray tool Partek to perform an analysis of variance and determine any significant fold changes (KD1, KD3, HC, and FC individually). Results: The data revealed that the AGO2 and AGO4 genes displayed significant within-group differences with *p* = 0.034 and 0.007, respectively. In AGO2, significant differences were observed between KD1 vs. HC + FC with *p* = 0.034. KD1 appears higher than the other specimens in AGO4, with significant differences between KD1 and HC (*p* = 0.033), KD1 and FC (*p* = 0.033), KD1 and KD3 (*p* = 0.013), and KD1 and HC + FC (*p* = 0.007). We observed no substantial differences in AGO1 or AGO3 (*p* > 0.05). There were no significant differences between AGO(s) and coronary artery lesions or intravenous immunoglobulin resistance. (*p* > 0.05) Conclusion: Endothelial cell inflammation and injury, two basic pathological mechanisms, are thought to be involved in coronary endothelial dysfunction in KD. AGO2 and AGO4 are likely to participate in the endothelial dysfunction of children with KD, with AGO4 potentially playing a key role, while AGO1 and AGO3 appear not to participate.

## 1. Introduction

Kawasaki disease (KD), which predominantly affects children under the age of 5 years old [1], is an acute systemic vasculitis that has the potential to result in coronary arteritis and a subsequent risk of coronary artery aneurysms [2]. While some researchers believe that KD activates the immune system via an infectious stimulus in a genetically susceptible child, the cause of KD remains undetermined [3]. KD is diagnosed when a patient presents with a high fever lasting five days or more and the appearance of at least four of the following five clinical criteria: strawberry tongue, bilateral conjunctivitis without discharge, hand and foot erythema, polymorphic rash, and cervical lymphadenopathy [4,5]. A KD diagnosis relies on identifying typical clinical features, but no diagnostic test is presently available for this disease [3,6]. Treating KD can be difficult due to its complex inflammatory etiology [7]. Therefore, researchers have recently aimed to determine the environmental and genetic factors that induce this excessive inflammatory response [8].

In KD, inflammation primarily targets the endothelial cells that line the blood vessels, including coronary arteries. Endothelial dysfunction has been correlated with a higher risk of small vessel disease [9,10], as well as identified as a predictor of hypertension [11] and other cardiovascular diseases like diabetes mellitus [12]. The immune system’s inflammatory response and vascular endothelial dysfunction are the primary reasons for coronary artery disease, which is a major complication of KD [13,14]. In particular, Chang et al. found that endothelial cell inflammation and injury form two fundamental pathological mechanisms, with endothelial cell pyroptosis potentially participating in coronary endothelial damage in KD [7]. Endothelial dysfunction likely develops before atherosclerotic plaque or coronary artery lesions appear during the early stage of KD [15,16,17,18].

Cells in the body must embrace distinct structures and create functionally definite subcellular compartments in order to carry out their daily biological functions. This process is achieved in part through an unequal distribution of messenger RNA (mRNA) within the cells [19]. According to a study by Suter et al., the localization of mRNA has become a vital part of the unequal distribution of proteins and protein complexes of an expanded size range [20]. The genetic information deposited in mRNA is translated with great accuracy into a protein sequence during mRNA translation [21]. Various diseases, particularly cardiovascular diseases, neurodegenerative diseases, and cancers, may result from masking and demasking mRNA disorders [22]. In endothelial cell dysfunction, mRNA expression plays a critical role in regulating various pathways that contribute to vascular damage, inflammation, and impaired barrier function. Changes in mRNA expression can alter the production of proteins involved in maintaining endothelial integrity, controlling inflammation, and regulating the coagulation system. Silvia et al. mentioned that a proportion of natriuretic peptide receptor-C mRNA was elevated in patients with impaired endothelial function [23], while reprogramming of mRNA translation via altered transfer RNA activity has been speculated to result in pathological processes in a codon-dependent way [24]. Furthermore, mRNA localization disruptions can severely affect developmental processes, which may then be partially responsible for various diseases [25]. On the other hand, studies have shown that mRNA has significant potential in preventing and treating numerous diseases and has thus emerged as an important focus for developing therapeutic agents [26].

Micro RNAs (miRNAs) modulate post-transcriptional gene expression. The biogenesis of these molecules involves numerous steps, and they are eventually loaded onto argonaute (AGO) proteins [27]. AGOs are greatly conserved and extensively expressed in almost all organisms [28]. Humans are found to have four AGO proteins (AGO1, 2, 3, and 4) that primarily display overlapping functions when loaded with specific miRNAs; however, each AGO has distinct roles under established conditions [29]. Argonaute proteins function as nucleic-acid-guided endonucleases, cleaving targets complementary to their DNA or RNA guides. This cleavage results in translational silencing, either directly or indirectly, by recruiting additional silencing proteins. Across and within different domains of life, argonaute proteins operate in distinct pathways, with the detection of mobile genetic elements (MGEs) triggering diverse immunity mechanisms. Evidence indicates that red blood cell (RBC)-derived miRNAs interact with the cardiovascular system through extracellular vesicles and argonaute RISC catalytic component 2 as carriers. Since AGOs are known for downgrading specific mRNAs and thus reducing the expression of a specific gene, elements from the varied AGO family serve various immune systems [30]. Accordingly, AGOs, which are inherently equipped with small single-stranded RNA or DNA, can likely discover and suppress complementary mobile genetic elements [30]. Researchers are currently studying which AGO primarily creates significant gene differences within a group. AGO proteins are central components of the RNA-induced silencing complex (RISC) and play a key role in regulating gene expression through interactions with miRNAs and small interfering RNAs (siRNAs). In endothelial cell dysfunction or cardiovascular diseases, the expression of AGO proteins and their involvement in miRNA-mediated gene silencing can significantly affect the regulation of pathways related to inflammation, vascular integrity, and oxidative stress. In this research, we focused on clarifying which AGO(s) are involved in the pathogenesis of KD.

## 2. Materials and Methods

### 2.1. Participants

This study included a total of 50 children with KD, 18 healthy children without any history of KD as healthy controls (HC), and another 18 febrile children who had a fever but were not diagnosed with KD as febrile controls (FC) for Human Transcriptome Array analysis (HTA 2.0). HC participants had no fever, infection, or KD history. The subjects in the FC set consisted of patients admitted to the hospital for lower airway infections, adenovirus, mycoplasma infection, respiratory syncytial-related viruses, herpangina, or gastroenteritis. All participating children with KD met the diagnostic criteria established by the American Heart Association (AHA). These criteria include a fever lasting 5 days or more and four out of five of the following: oral mucosal inflammation, bilateral non-suppurative conjunctivitis, cervical lymph node enlargement, erythema or edema of the palms and soles, and polymorphous rash [31]. Every child with KD was given one or two doses of IVIG 2 g/kg (body weight), which were infused over 12 h in accordance with current standards [31]. IVIG resistance or refractory KD is defined as persistent fever 48 h after the first IVIG infusion. A second IVIG infusion (2 g/kg for 12 h) was only administered in the case of refractory KD. We obtained blood specimens from children with KD twice: within 24 h before IVIG therapy (KD1, KD before IVIG infusion) and at least 3 weeks following IVIG therapy (KD3, KD after IVIG infusion). Coronary artery lesions (CAL) are defined as the inside width of the coronary arteries having a z-score ≥ 2.5 or the actual evaluation of the coronary arteries being greater than 3 mm for those younger than 5 years old or greater than 4 mm for those older than 5 years old [32]. The hospital’s Institutional Review Board provided its consent for this research (IRB No. 102-3779A3 with the ethical approval date on 22 October 2012), and we also obtained the written informed consent of the parents or guardians of every child with KD included in this study.

**Figure 1:**
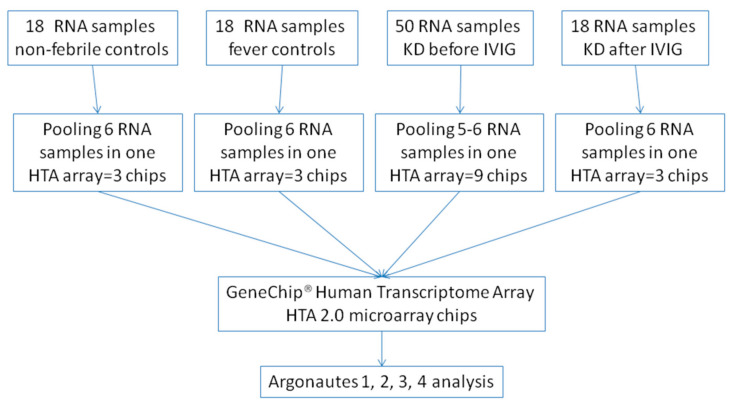
Flowchart of study.

We collected whole blood samples from the subjects and performed red blood cell (RBC) lysis using an RBC lysis buffer to enrich samples for total white blood cells (WBCs). We then used the mirVana™ miRNA Isolation Kit (Ambion, Foster City, CA, USA) to extract RNA, following the manufacturer’s protocols as described in a previous study. RNA concentrations were measured with the NanoDrop 2000 spectrophotometer (Thermo Scientific, Waltham, MA, USA). All RNA samples met the criterion of a RIN ≥ 7, as assessed by the Agilent 2100 Bioanalyzer (Agilent, Santa Clara, CA, USA). The collected RNA samples were then subjected to a microarray assay to determine the gene expression profile. In this study, we used Affymetrix HTA 2.0 microarray chips for profiling (HTA 2.0, Affymetrix, Santa Clara, CA, USA). The RNA samples were first prepared using the WT PLUS Reagent Kit (Affymetrix), followed by hybridization on the HTA 2.0 microarray chips. We obtained mRNA-level gene expression profiling from leukocyte samples that were gathered by a previous study and uploaded to the NCBI GEO database (GSE109351 series). In total, the analysis for Human Transcriptome Array included 50 children with KD prior to IVIG infusion, 18 children with KD three weeks post-IVIG therapy, 18 healthy (without any fever or infection) controls, and 18 febrile controls (see Table 1) during the period of 2012–2014. Data for all methods and materials are provided in the aforementioned publications. Each sample was pooled from six patients. We used the recently created KD markers internet tool (https://cosbi.ee.ncku.edu.tw/KDmarkers/, accessed on 13 January 2022) to evaluate the expression of AGO1, 2, 3, and 4 genes in the leukocytes of children with KD. This internet server has gene expression data from the GeneChip Human Transcriptome Array 2.0 (Thermo Fisher Scientific Inc., Waltham, MA, USA). The raw data from the HTA 2.0 chips were first subjected to quality control examination according to the guidelines in the Affymetrix manuals. The chips that passed the quality control criteria were then analyzed using the commercialized microarray tool Partek (Partek Inc., St Louis, MO, USA). We performed an analysis of variance using the default value of Partek to determine any significant fold changes (KD1, KD3, HC, and FC individually divided by healthy controls) (Figure 1).

### 2.2. Gene Expression Profiling with Microarray (HTA 2.0)

For strong, unbiased results, we created RNA libraries by evenly pooling 5–6 RNA samples; we obtained three pooled normal control libraries (3 arrays from 18 cases with the 6 RNA pooling method), three fever control libraries (3 arrays from 18 cases with the 6 RNA pooling method), nine pre-IVIG KD libraries (from 50 KD samples), and three post-IVIG KD (from 18 KD samples) libraries. Among 50 patients with KD, 32 cases can identify 14 with CAL formation and 9 with IVIG resistance (not mixed, pooled). We submitted these pooled RNA samples for microarray assays to establish gene expression profiles. Further profiling was performed using the GeneChip^®^ Human Transcriptome Array. We prepared RNA samples with the WT PLUS Reagent kit and then hybridized them on HTA 2.0 microarray chips. In accordance with the Affymetrix instruction manual, the raw data from the HTA 2.0 chips underwent quality control examination as previously described [2]. The chips that passed the quality control criteria were analyzed using Partek Genomic Suite 6.4 (Partek, St. Louis, MO, USA), a commercial software designed specifically for microarray data analysis. We also used Partek to conduct an ANOVA and report *p*-values for the comparisons of interest.

### 2.3. Statistics

Every value in this study is shown as mean ± standard error (SE). Once a chip passed the quality assurance guide, we assessed it using Partek [2]. Quantitative data were examined using the Kruskal–Wallis H test, Mann–Whitney *U* test, or one-way ANOVA as appropriate. We used SPSS version 14.0 (SPSS, Inc., Chicago, IL, USA) to perform statistical analysis, and a two-sided *p*-value less than 0.05 was considered statistically significant.

## 3. Results

**Figure 2:**
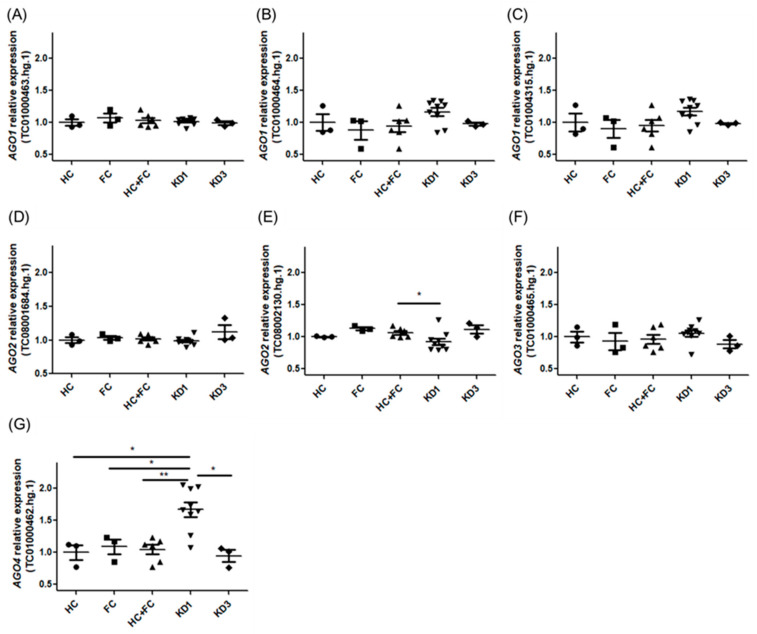
Expression analysis of AGO1 ((**A**): TC01000463.hg.1; (**B**): TC01000464.hg.1; and (**C**): TC01004315.hg.1), AGO2 ((**D**): TC08001684.hg.1; and ((**E**): TC08002130.hg.1), AGO3 ((**F**): TC01000465.hg.1), and AGO4 ((**G**): TC01000462.hg.1) in the leukocytes of Kawasaki disease patients and controls. HC, non-febrile (healthy) control; FC, febrile control; KD1, children with KD within 24 h before IVIG (intravenous immunoglobulin) infusion; KD3, children with KD at least three weeks post-IVIG infusion. (ANOVA, analyses of variance). * indicated *p* < 0.05, ** indicated *p* < 0.01.

This study consists of specimens from febrile controls (*n* = 18), healthy controls (*n* = 18), overall controls (febrile and healthy controls, *n* = 36), patients with KD1 (children with KD within 24 h prior to IVIG infusion, *n* = 50), and patients with KD3 (children with KD at least three weeks post-IVIG infusion, *n* = 18) (demographic data shown in Table 1). There were 10 patients with IVIG resistance while 20 patients with KD with CAL formation in 50 patients with KD. These four groups were designed to evaluate the AGO1, AGO2, AGO3, and AGO4 expressions to determine whether significant differences could be detected within any genes. The gene expression of AGO included TC01000463.hg.1, TC01000464.hg.1 and TC01004315.hg.1 for AGO1; TC08001684.hg.1 and TC08002130.hg.1 for AGO2; TC01000465.hg.1 for AGO3; and TC01000462.hg.1 for AGO4 (Table 2). The results revealed significant differences in both the AGO2 and AGO4 genes (Figure 2 and Table 3). In AGO2, we found significant differences between KD1 and HC + FC (0.93 ± 0.15 vs. 1.06 ± 0.08, *p* = 0.034, fold change 0.92). KD1 is apparently higher than the other specimens in AGO4, and we observed significant differences between KD1 and HC (1.67 ± 0.34 vs. 1.00 ± 0.20, *p* = 0.033, fold change 1.67), KD1 and FC (1.67 ± 0.34 vs. 1.09 ± 0.21, *p* = 0.033, fold change 1.54), KD1 and KD3 (1.67 ± 0.34 vs. 0.95 ± 0.16, *p* = 0.013, fold change 1.76), and KD1 and HC + FC (1.67 ± 0.34 vs. 1.04 ± 0.19, *p* = 0.007, fold change 1.51). However, AGO1 and AGO3 showed no significant differences among those groups (all *p* > 0.05). There were no significant differences between AGO(s) expression with CAL formation (*p* > 0.05) and IVIG resistance (*p* > 0.05). (Supplementary Tables S1 and S2).

## 4. Discussion

Kawasaki disease (KD) is a systemic vasculitis characterized by endothelial cell dysfunction. Immuno-inflammatory response and vascular endothelial dysfunction are key contributors to coronary artery disease in patients with Kawasaki disease (KD). While no specific pathogens or pathogen-related structural substances have been directly linked to KD, the disease is presumed to be triggered by an abnormal inflammatory response to infection in genetically susceptible individuals. Semaphorin 7A (Sema7A) has been reported to regulate endothelial phenotypes associated with cardiovascular diseases in KD. A recent report showed that Sema7A contributes to the progression of KD vasculitis by promoting endothelial permeability and inflammation through a Plexin C1- and Integrin β1-dependent pathway.

In line with a molecular medicine approach, evaluating the expression, creation, liberation, and roles of peptides can provide helpful information for a more thorough interpretation of a disease’s mechanism and complications [23]. Small RNAs are indispensable for various basic life processes. AGO proteins are connected with the abundant types of small RNAs and thus are also essential in small RNA-moderated regulatory pathways [33]. Plants transfer small RNAs into their fungal pathogens via extracellular vesicles (EVs) and suppress genes involved in fungal transmission; i.e., small RNAs are selectively loaded into EVs [34]. In arabidopsis, the proteins RH11, RH37, and AGO1 restrictively tie to EV-filled small RNAs instead of non-EV-connected small RNAs, indicating that these proteins enhance the meticulous piling of small RNAs into EVs [34]. In humans, AGO proteins play vital roles by attaching to small RNAs and modulating their targets’ expressions. This phenomenon occurs when the miRNA pathway represses genes to promote decomposition and translational suppression, while the siRNA pathway suppresses transcripts via direct AGO2-moderated cleavage [35]. In recent years, studies have discovered that most miRNAs operate repetitively across the four AGO proteins [29]. Among the AGOs, AGO2 has consistently been considered the exemplified paralog, but increasing evidence has shown that the other AGOs also play unique roles in various biological processes and diseases [29].

Kuo et al. previously reported that miRNAs likely participate in gene regulation and mediate communication between cells far away from each other, as well as that some miRNAs discovered in the serum may regulate the efficacy of vascular endothelial cells in patients with KD [4]. miRNAs participate in gene expression regulation by interfering with the translation of mRNA into proteins [4]. While the combination of certain AGO and miRNA complexes may be adequate to recognize specific RNAs, the induction of effector proteins assists in regulating biological function [36]. Liao et al. verified that the overexpression of both miRNA-19a-3p and miRNA-184 target AGO2, resulting in endothelial dysfunction in KD [37]. Another study [29] reported that, due to a lack of a sifting system in humans, miRNAs are casually incorporated into the four AGO proteins based on their populations in the cell, with the exception of AGO4, which is known to have a specialized target specificity. Furthermore, siRNA reduces the methylation level of a specific miRNA by silencing AGO4, but not AGO1, AGO2, or AGO3. These factors may be the main reasons for AGO4’s unique action of inducing endothelial dysfunction, but it may also be that more than one pathway is causing this dysfunction in KD or that different miRNAs are inducing different routes by attacking dissimilar AGOs. Our findings suggest that AGO2 and AGO4 are involved in inducing endothelial dysfunction in KD, but AGO1 and AGO3 are not. Among these AGOs, AGO4 may play a primary role with significant elevation during the early stage of KD, which then subsides following IVIG treatment.

Human transcriptomes are composed of non-coding RNAs, and some researchers have speculated an intertwined interplay among varied RNA species, including non-coding RNAs and mRNAs [38]. This innovative RNA crosstalk likely contributes to gene regulatory networks and has implications in both human development and diseases [38]. In recent years, some studies have found that miRNAs in serum exosomes or coronary artery tissues, including miR-93, miR-186, miR-223, miR-483, and miR-23a, are associated with KD [39,40]. Whether an intricate interaction among diverse RNA species or various types of RNA transcripts are involved in endogenous RNAs remains unknown, which may explain why our study has different outcomes related to AGOs compared to a previous study.

Although KD diagnosis of a patient is generally made with a persistent high fever and the appearance of five clinical traits, as shown in previous studies [4,5], with headache, extreme irritability, and somnolence [41] presenting in early and/or primary manifestations in some children with KD, these are only clinical features of the disease, not confirmatory tests for diagnosis. Relying on clinical features alone may result in the misdiagnosis, delayed diagnosis, or under-diagnosis of KD, especially for patients who do not present typical clinical characteristics. All of the above scenarios may enhance the risk of coronary artery lesions. Establishing novel markers, like miRNAs, eosinophil levels, and diagnosis techniques [4], together with clinical features, can help practicing physicians rapidly recognize children with KD. We hope that this article can contribute to developing approaches for quick KD recognition and thus reduce coronary artery abnormalities in children.

The mechanistic specialization of AGO2 (directly cleaving mRNAs of key inflammatory regulators and selectively binding miRNAs that modulate vascular inflammatory pathways) and AGO4 (epigenetically modulating gene expression through chromatin remodeling and transcriptional silencing) makes them pivotal in driving vascular inflammation. In contrast, AGO1 and AGO3 lack catalytic and epigenetic regulatory capacities and have broader, less specific RNA silencing roles and thus contribute minimally to inflammatory amplification.

Targeting AGO2 and AGO4 offers promising therapeutic potential for managing vascular inflammation due to their distinct mechanistic roles. AGO2, with its catalytic slicer activity, regulates inflammation by cleaving mRNAs of key inflammatory mediators and binding pro-inflammatory miRNAs, such as miR-155 and miR-21. Future therapies could potentially inhibit AGO2’s activity, modulate its miRNA interactions, or enhance the function of anti-inflammatory miRNAs, such as miR-146a. On the other hand, AGO4 influences inflammation through epigenetic mechanisms, including chromatin remodeling and transcriptional silencing of anti-inflammatory genes. Therapies that target AGO4 could involve disrupting its interactions with the siRNAs guiding these modifications or combining it with epigenetic drugs like HDAC or DNMT inhibitors. Potential applications include treating atherosclerosis, autoimmune vasculitis, and chronic inflammatory vascular conditions, but such challenges as off-target effects and long-term safety would need to be addressed.

This study has several limitations that should be noted. At first, this is a single-center study. Then there are no data on AGO gene expression in between different age and sex groups, different levels of inflammation of different etiologies, and pretreatment before the samples were collected. We did not investigate the comprehensive route of how miRNAs and hub genes modulate KD deficiency. Second, we did not validate the outcomes of gene expression profiling with microarray performed using bioinformatics analysis with laboratory experiments. In the future, studies should include practical strategies for thorough clinical evaluation, e.g., using such methods as real-time PCR, Western blotting, ELISA, etc., in order to validate the gene and protein levels of AGOs, as well as to verify and improve the sensitivity and specificity of biomarkers. Future studies should also increase patient numbers.

## 5. Conclusions

Researchers believe that the two basic pathological mechanisms of endothelial cell injury and inflammation are involved in coronary artery endothelial damage in KD. AGO2 and AGO4 likely participate in the endothelial cell injury of children with KD, with AGO4 potentially playing a key role, while AGO1 and AGO3 appear not to participate. By influencing these pathways of modulating expression, AGO4-targeted therapies may help restore endothelial health and prevent the progression of vascular diseases.

## Figures and Tables

**Table 1:** Demographic characteristics of 104 patients.

	Non-Febrile Controls (*n* = 18)			Febrile Controls (*n* = 18)			KD Before IVIG (*n* = 50)			KD After IVIG (*n* = 18)			*p* Value
Gender, Male, N (%)	9.0		(50.0)	8.0		(44.4)	27.0		(54.0)	9.0		(50.0)	0.918
Age, years	2.46	±	1.50	1.97	±	1.09	1.36	±	1.00	1.74	±	1.27	0.003 **
CRP (mg/L)	N/A			27.48	±	27.48	87.08	±	67.21	1.15	±	2.30	*p* < 0.001
WBC (1000/uL)	8.98	±	2.18	8.86	±	3.17	14.96	±	7.40	8.67	±	2.06	*p* < 0.001
Hemoglobin (g/dL)	12.67	±	0.60	12.09	±	1.14	10.97	±	0.93	11.91	±	0.84	*p* < 0.001
Platelets(1000/uL)	302.39	±	101.77	291.56	±	118.99	359.82	±	156.46	362.24	±	61.76	0.023 *

* indicates *p* < 0.05 and ** indicates *p* < 0.01 between the groups. Data were presented in mean ± SD. Differences between groups were analyzed using the Kruskal–Wallis H test. KD: Kawasaki disease; IVIG: intravenous immunoglobulin; N/A: no detection; CRP: C-reactive protein; WBC: white blood cell.

**Table 2:** Gene symbol for AGO expression.

Gene Symbol	Column ID	HC (*n* = 3)	FC (*n* = 3)	KD1 (*n* = 9)	KD3 (*n* = 3)	HC + FC (*n* = 6)
AGO1	TC01000463.hg.1	1.00 ± 0.09	1.07 ± 0.12	1.02 ± 0.05	0.99 ± 0.05	1.03 ± 0.10
AGO1	TC01000464.hg.1	1.00 ± 0.23	0.88 ± 0.25	1.17 ± 0.19	0.98 ± 0.04	0.94 ± 0.23
AGO1	TC01004315.hg.1	1.00 ± 0.24	0.90 ± 0.25	1.17 ± 0.18	0.98 ± 0.02	0.95 ± 0.23
AGO2	TC08001684.hg.1	1.00 ± 0.07	1.03 ± 0.05	0.98 ± 0.06	1.12 ± 0.18	1.02 ± 0.06
AGO2	TC08002130.hg.1	1.00 ± 0.01	1.13 ± 0.04	0.93 ± 0.15	1.11 ± 0.11	1.06 ± 0.08
AGO3	TC01000465.hg.1	1.00 ± 0.15	0.93 ± 0.23	1.05 ± 0.15	0.89 ± 0.12	0.96 ± 0.18
AGO4	TC01000462.hg.1	1.00 ± 0.20	1.09 ± 0.21	1.67 ± 0.34	0.95 ± 0.16	1.04 ± 0.19

AGO: argonautes; KD1: Kawasaki disease within 24 h before IVIG treatment; KD3: Kawasaki disease at least 3 weeks after IVIG treatment; FC: febrile control; HC: non-febrile control. All values are expressed as mean ± standard error (SE).

**Table 3:** mRNA expressions of AGO between Kawasaki disease patients and control subjects.

Gene Symbol	Column ID	Fold-Change (KD1 vs. HC)	*p*-Value (KD1 vs. HC)	Fold-Change (KD1 vs. FC)	*p*-Value (KD1 vs. FC)	Fold-Change (KD1 vs. KD3)	*p*-Value (KD1 vs. KD3)	Fold-Change (KD1 vs. HC + FC)	*p*-Value (KD1 vs. HC + FC)
AGO1	TC01000463.hg.1	1.02	0.644	1.30	0.405	1.19	0.518	1.30	0.814
AGO1	TC01000464.hg.1	1.17	0.309	0.95	0.079	1.02	0.166	1.06	0.077
AGO1	TC01004315.hg.1	1.17	0.229	1.33	0.079	1.19	0.116	1.31	0.059
AGO2	TC08001684.hg.1	0.98	0.926	0.95	0.166	0.88	0.052	1.05	0.346
AGO2	TC08002130.hg.1	0.93	0.166	0.82	0.052	0.83	0.079	0.92	0.034 *
AGO3	TC01000465.hg.1	1.05	0.405	1.14	0.518	1.19	0.079	1.06	0.346
AGO4	TC01000462.hg.1	1.67	0.033 *	1.54	0.033 *	1.76	0.013 *	1.51	0.007 **

* Indicates *p* < 0.05; ** indicates *p* < 0.01. KD1: Kawasaki disease within 24 h before IVIG treatment; KD3: Kawasaki disease at least 3 weeks after IVIG treatment; FC: febrile control; HC: non-febrile control. (Mann–Whitney U test).

## Data Availability

The data collection analyzed during the present research are available from the corresponding author upon fair request.

## Supplementary Materials

The following supporting information can be downloaded at https://www.mdpi.com/article/10.3390/children12010073/s1: Table S1: AGO mRNA expression of the without CAL and with CAL groups; Table S2: AGO mRNA expression of the IVIG responsive and IVIG resistance groups.

## References

[1] Qiu Y., Zhang Y., Li Y., Hua Y., Zhang Y. (2022). Molecular mechanisms of endothelial dysfunction in Kawasaki-disease-associated vasculitis. Front. Cardiovasc. Med..

[2] Huang Y.H., Lo M.H., Cai X.Y., Liu S.F., Kuo H.C. (2019). Increase expression of CD177 in Kawasaki disease. Pediatr. Rheumatol. Online J..

[3] Scuccimarri R. (2012). Kawasaki disease. Pediatr. Clin. N. Am..

[4] Kuo H.C. (2023). Diagnosis, Progress, and Treatment Update of Kawasaki Disease. Int. J. Mol. Sci..

[5] Messenger N., Messenger G., Potts G. (2023). Pustular rash masking Kawasaki disease. Int. J. Dermatol..

[6] Jindal A.K., Pilania R.K., Prithvi A., Guleria S., Singh S. (2019). Kawasaki disease: Characteristics, diagnosis, and unusual presentations. Expert Rev. Clin. Immunol..

[7] Jia C., Zhang J., Chen H., Zhuge Y., Chen H., Qian F., Zhou K., Niu C., Wang F., Qiu H. (2019). Endothelial cell pyroptosis plays an important role in Kawasaki disease via HMGB1/RAGE/cathespin B signaling pathway and NLRP3 inflammasome activation. Cell Death Dis..

[8] Tirelli F., Marrani E., Giani T., Cimaz R. (2020). One year in review: Kawasaki disease. Curr. Opin. Rheumatol..

[9] Balta S. (2021). Endothelial Dysfunction and Inflammatory Markers of Vascular Disease. Curr. Vasc. Pharmacol..

[10] Wardlaw J.M., Smith C., Dichgans M. (2019). Small vessel disease: Mechanisms and clinical implications. Lancet Neurol..

[11] Caballero-Eraso C., Munoz-Hernandez R., Asensio Cruz M.I., Moreno Luna R., Carmona Bernal C., López-Campos J.L., Stiefel P., Sanchez Armengol A. (2019). Relationship between the endothelial dysfunction and the expression of the β1-subunit of BK channels in a non-hypertensive sleep apnea group. PLoS ONE.

[12] Jin J., Wang X., Zhi X., Meng D. (2019). Epigenetic regulation in diabetic vascular complications. J. Mol. Endocrinol..

[13] Koibuchi H., Kotani K., Minami T., Konno K., Taniguchi N. (2016). Endothelial dysfunction by flow-mediated dilation assessed ultrasonically in patients with Kawasaki Disease. Minerva Pediatr..

[14] Wang Y., Li T. (2022). Advances in understanding Kawasaki disease-related immuno-inflammatory response and vascular endothelial dysfunction. Pediatr. Investig..

[15] Gao M., Yu T., Liu D., Shi Y., Yang P., Zhang J., Wang J., Liu Y., Zhang X. (2021). Sepsis plasma-derived exosomal miR-1-3p induces endothelial cell dysfunction by targeting SERP1. Clin. Sci..

[16] Xiao X., Xu M., Yu H., Wang L., Li X., Rak J., Wang S., Zhao R.C. (2021). Mesenchymal stem cell-derived small extracellular vesicles mitigate oxidative stress-induced senescence in endothelial cells via regulation of miR-146a/Src. Signal Transduct. Target. Ther..

[17] Desantis V., Potenza M.A., Sgarra L., Nacci C., Scaringella A., Cicco S., Solimando A.G., Vacca A., Montagnani M. (2023). microRNAs as Biomarkers of Endothelial Dysfunction and Therapeutic Target in the Pathogenesis of Atrial Fibrillation. Int. J. Mol. Sci..

[18] Routhu S.K., Singhal M., Jindal A.K., Kumar V., Yadav A.K., Singh S. (2021). Assessment of Endothelial Dysfunction in Acute and Convalescent Phases of Kawasaki Disease Using Automated Edge Detection Software: A Preliminary Study from North India. J. Clin. Rheumatol..

[19] Loedige I., Baranovskii A., Mendonsa S., Dantsuji S., Popitsch N., Breimann L., Zerna N., Cherepanov V., Milek M., Ameres S. (2023). mRNA stability and m(6)A are major determinants of subcellular mRNA localization in neurons. Mol. Cell.

[20] Suter B. (2018). RNA localization and transport. Biochim. Biophys. Acta Gene Regul. Mech..

[21] Sonneveld S., Verhagen B.M.P., Tanenbaum M.E. (2020). Heterogeneity in mRNA Translation. Trends Cell Biol..

[22] Voronina A.S., Pshennikova E.S. (2021). mRNPs: Structure and role in development. Cell Biochem. Funct..

[23] Del Ry S., Cabiati M., Bianchi V., Randazzo E., Peroni D., Clerico A., Federico G. (2020). C-type natriuretic peptide plasma levels and whole blood mRNA expression show different trends in adolescents with different degree of endothelial dysfunction. Peptides.

[24] Orellana E.A., Siegal E., Gregory R.I. (2022). tRNA dysregulation and disease. Nat. Rev. Genet..

[25] Otis J.P., Mowry K.L. (2023). Hitting the mark: Localization of mRNA and biomolecular condensates in health and disease. Wiley Interdiscip. Rev. RNA.

[26] Zhou D.W., Wang K., Zhang Y.A., Ma K., Yang X.C., Li Z.Y., Yu S.S., Chen K.Z., Qiao S.L. (2023). mRNA therapeutics for disease therapy: Principles, delivery, and clinical translation. J. Mater. Chem. B.

[27] Matsuyama H., Suzuki H.I. (2019). Systems and Synthetic microRNA Biology: From Biogenesis to Disease Pathogenesis. Int. J. Mol. Sci..

[28] Jin S., Zhan J., Zhou Y. (2021). Argonaute proteins: Structures and their endonuclease activity. Mol. Biol. Rep..

[29] Nakanishi K. (2022). Anatomy of four human Argonaute proteins. Nucleic Acids Res..

[30] Bobadilla Ugarte P., Barendse P., Swarts D.C. (2023). Argonaute proteins confer immunity in all domains of life. Curr. Opin. Microbiol..

[31] McCrindle B.W., Rowley A.H., Newburger J.W., Burns J.C., Bolger A.F., Gewitz M., Baker A.L., Jackson M.A., Takahashi M., Shah P.B. (2017). Diagnosis, Treatment, and Long-Term Management of Kawasaki Disease: A Scientific Statement for Health Professionals from the American Heart Association. Circulation.

[32] (2010). Guidelines for diagnosis and management of cardiovascular sequelae in Kawasaki disease (JCS 2008)–digest version. Circ. J..

[33] Zhai L., Wang L., Teng F., Zhou L., Zhang W., Xiao J., Liu Y., Deng W. (2016). Argonaute and Argonaute-Bound Small RNAs in Stem Cells. Int. J. Mol. Sci..

[34] He B., Cai Q., Qiao L., Huang C.Y., Wang S., Miao W., Ha T., Wang Y., Jin H. (2021). RNA-binding proteins contribute to small RNA loading in plant extracellular vesicles. Nat. Plants.

[35] Sala L., Chandrasekhar S., Vidigal J.A. (2020). AGO unchained: Canonical and non-canonical roles of Argonaute proteins in mammals. Front. Biosci..

[36] Johnson K.C., Johnson S.T., Liu J., Chu Y., Arana C., Han Y., Wang T., Corey D.R. (2023). Consequences of depleting TNRC6, AGO, and DROSHA proteins on expression of microRNAs. RNA.

[37] Liao J., Guo X., Fan X., Zhang X., Xu M. (2023). Upregulation of miR-184 and miR-19a-3p induces endothelial dysfunction by targeting AGO2 in Kawasaki disease. Cardiol. Young.

[38] Tay Y., Rinn J., Pandolfi P.P. (2014). The multilayered complexity of ceRNA crosstalk and competition. Nature.

[39] Saito K., Nakaoka H., Takasaki I., Hirono K., Yamamoto S., Kinoshita K., Miyao N., Ibuki K., Ozawa S., Watanabe K. (2016). MicroRNA-93 may control vascular endothelial growth factor A in circulating peripheral blood mononuclear cells in acute Kawasaki disease. Pediatr. Res..

[40] Wu R., Shen D., Sohun H., Ge D., Chen X., Wang X., Chen R., Wu Y., Zeng J., Rong X. (2018). miR-186, a serum microRNA, induces endothelial cell apoptosis by targeting SMAD6 in Kawasaki disease. Int. J. Mol. Med..

[41] Liu X., Zhou K., Hua Y., Wu M., Liu L., Shao S., Wang C. (2020). Neurological involvement in Kawasaki disease: A retrospective study. Pediatr. Rheumatol. Online J..

