# Supplementary material for: Argonaute2 and Argonaute4 Involved in the Pathogenesis of Kawasaki Disease via mRNA Expression Profiles
Source: Children (Basel). 2025 Jan 8;12(1):73. doi: 10.3390/children12010073 (PMC11763442; doi:10.3390/children12010073)
Supplement: Supplementary file 1 [file children-12-00073-s001.zip › children-3361957-supplementary.pdf]

Supplementary Table S1. AGO mRNA expression of the without CAL and with CAL groups.

AGO mRNA expression	Without CAL (N=18)			With CAL (N=14)			p value
AGO1_TC01000463.hg.1	1.18	±	0.13	1.18	±	0.19	0.488
AGO1_TC01000464.hg.1	1.04	±	0.01	1.00	±	0.04	0.085
AGO1_TC01004315.hg.1	1.20	±	0.12	1.14	±	0.24	0.160
AGO2_TC08001684.hg.1	0.97	±	0.03	0.95	±	0.06	0.281
AGO2_TC08002130.hg.1	0.87	±	0.07	0.83	±	0.06	0.601
AGO3_TC01000465.hg.1	1.04	±	0.03	1.15	±	0.11	0.238
AGO4_TC01000462.hg.1	1.78	±	0.22	1.59	±	0.49	0.312

Supplementary Table S2. AGO mRNA expression of the IVIG responsive and IVIG resistance groups.

AGO mRNA expression	IVIG responsive (N=23)			IVIG resistance (N=9)			p value
AGO1_TC01000463.hg.1	1.16	±	0.16	1.22	±	0.17	0.800
AGO1_TC01000464.hg.1	1.02	±	0.03	1.02	±	0.05	0.800
AGO1_TC01004315.hg.1	1.15	±	0.20	1.21	±	0.14	1.000
AGO2_TC08001684.hg.1	0.96	±	0.05	0.95	±	0.02	0.533
AGO2_TC08002130.hg.1	0.87	±	0.06	0.80	±	0.01	0.267
AGO3_TC01000465.hg.1	1.09	±	0.12	1.11	±	0.06	0.533
AGO4_TC01000462.hg.1	1.51	±	0.30	2.04	±	0.02	0.133
